# Autophagy as a Vital Therapy Target for Renal Cell Carcinoma

**DOI:** 10.3389/fphar.2020.518225

**Published:** 2021-02-10

**Authors:** Ying-hua He, Guo Tian

**Affiliations:** ^1^ Department of Clinical Pharmacy, The First Affiliated Hospital, College of Medicine, Zhejiang University, Hangzhou, China; ^2^ Hepatobiliary and Pancreatic Intervention Center, The First Affiliated Hospital, College of Medicine, Zhejiang University, Hangzhou, China

**Keywords:** autophagy, rcc, inhibitor, inducers, PI3K/AKT/mTOR, AMPK/mTOR

## Abstract

Autophagy is a process that degrades and recycles superfluous organelles or damaged cellular contents. It has been found to have dual functions in renal cell carcinoma (RCC). Many autophagy-related proteins are regarded as prognostic markers of RCC. Researchers have attempted to explore synthetic and phytochemical drugs for RCC therapy that target autophagy. In this review, we highlight the importance of autophagy in RCC and potential treatments related to autophagy.

## Background

Renal cell carcinoma (RCC) is the 14th most common type of cancer globally, which comprises about 80 to 90% of malignant renal tumors (Hancock and Georgiades, 2016). Its incidence has been increasing at a rate of about 2% annually, with more than 200,000 new cases worldwide per year (Siegel et al., 2019). Despite advances in surgical techniques, the overall 5-year survival rate of patients in early-stage RCC is approximately 93%, whereas that of patients with metastatic RCC is approximately12% (Siegel et al., 2017; Attalla et al., 2020). Therefore, it is imperative to develop novel targets against RCC. Nevertheless, many studies have recently reported intimate relationships between autophagy and RCC, which may offer new options for the treatment of RCC.

Based on the different transport modes of intracellular components to the lysosome, autophagy could be categorized into three subtypes: macroautophagy, microautophagy, and chaperone-mediated autophagy (CMA) (Jacob et al., 2017). Macroautophagy is the most common subtype. The process of autophagy could be divided into four stages: initiation, elongation and completion, maturation, and fusion or degradation (Limpert et al., 2018; Cao and Bai, 2019). These ultrastructural studies indicate that at the autophagosome initiation stage, inhibition of mTOR induced the formation of ATG1/Unc-51-like kinases (ULK) complex (ULK1/2, ATG13, FIP200, and ATG101). This complex activates phosphatidylinositol 3-phosphate (PI3P) production through class III phosphatidylinositol 3-kinase complex (VPS34, ATG14 L, VPS15, and Beclin1). The production of PI3P recruits certain effectors, including proteins like double FYVE-containing protein 1 (DFCP1), and WD-repeat protein interacting with phosphoinositides (WIPIs) to form omegasomes (nucleation sites) (Mizushima et al., 2011; Devereaux et al., 2013). Possible sources of the autophagosomal membrane may include the endoplasmic reticulum (ER), mitochondria, Golgi apparatus, plasma membrane, and recycling endosomes (Karanasios et al., 2016). At the elongation and completion stage, the vesicle is further extended, curved, and closed to form the autophagosome, which is a double-layer membrane structure with approximately 500 to 1,500 nm diameter (Hale et al., 2013). This process requires two pivotal ubiquitin-like conjugation systems such as the ATG5–ATG12 complex, which conjugates with ATG16L1, and the microtubule-associated protein 1 light chain 3 (MAP1LC3, commonly called LC3), which conjugates with the lipid phosphatidylethanolamine (PE), commonly referred to as membrane-bound LC3-II (Klionsky and Schulman, 2014). During autophagy, LC3-I (LC3 precursor) undergoes cleavage and lipidation to form LC3-II, which is a crucial component of autophagosomes (Ogata et al., 2006). Next, LC3-II is recruited to the two sides of the autophagosome and acts as a receptor, which interacts with adaptor proteins like p62/SQSTM1 (Hirano et al., 2016). At the maturation stage, the autophagosome fuses with the lysosome to form an autolysosome (Noda and Inagaki, 2015). Lastly, at the fusion or degradation stage, the intracellular components are degraded and released into the cytosol (Tanida et al., 2005).

It is worth noting that research on the relationship between autophagy and RCC has become a subject of interest. Previous studies have established that autophagy-related proteins could act as promising prognostic biomarkers for the treatment of RCC (Q. Deng et al., 2018; Guo et al., 2019; Nishikawa et al., 2015). Bray et al. found that human RCCs have high basal autophagy which is required for survival to mTOR inhibition (Bray et al., 2012). It is attractive for autophagy inducers or inhibitors for the treatment of RCC. Therefore, this study aims to explore the molecular mechanisms of autophagy, focusing on the systematic analysis of autophagy in RCC.

### Autophagy-Related Proteins in RCC

Previously it was observed that Beclin1 is involved in assembling Beclin 1-Vps34-Vps15 core complexes and inducing autophagy, particularly under unfavorable conditions (R. Kang et al., 2011). Additionally, Bcl-2 inhibited autophagy through binding to Beclin1 (Grimaldi et al., 2015). Moreover, univariate and multivariate analysis showed that the expression level of Beclin1 appeared to be negatively correlated to the recurrence-free survival (RFS) in 100 patients with non-metastatic renal clear cell carcinoma (ccRCC) (Nishikawa et al., 2015). The high expression of Beclin 1 was also identified in tissues and cells of RCC (A498 and ACHN cell lines) (Guo et al., 2019).

The increased conversion of LC3-I to LC3-II is considered a marker for the initiation of autophagy because of its aggregation and localization on autophagosomes (Ogata et al., 2006). This research has revealed that cell mobility in ccRCC, A498, and ACHN cell lines is promoted by the up-regulated expression of LC3 (Guo et al., 2019). Two reports showed that promoting autophagy-related apoptosis resulted in down-regulation of LC3-II levels in RCC tissues and cells (Grimaldi et al., 2015; M. L.; Li et al., 2018). By contrast, Wang et al. found that LC3-II expression levels in RCC cell lines (786-O, 769-P, OS-RC-2, ACHN cells) were lower than those in a control cell line (HK-2 cell) (Wang et al., 2018).

Importantly, low expression of ATGs that is related to the process of autophagy nucleation, predicts poor prognosis in RCC (X. D. Liu et al., 2015a; Yu et al., 2018). Liu et al. found that most ccRCCs harbor allelic loss and/or mutation of ATG7. The efficient ATG7 down-regulation suppressing autophagy in RCC cell lines was evidenced by the down-regulation of LC3-II (Q. Deng et al., 2018; Wang et al., 2018). Studies have confirmed that the expression levels of ATG5-ATG12 conjugates is positively correlated with LC3-II aggregation and Beclin1 expression in RCC (M. L. Li et al., 2018; Turcotte et al., 2008).

P62/SQSTM1, a classical macroautophagy substrate, binds directly to LC3 to degrade ubiquitinated protein (Pankiv et al., 2007). Autophagy decreased P62 levels in RCC (Wu et al., 2015). Furthermore, p62 amplification on chromosome 5q was linked to renal cancer tumorigenesis (Mathew et al., 2009).

### Autophagy-Related Tumor Suppressor Genes in RCC

Research has shown that genetic dysregulation of autophagy is a key characteristic of different subtypes of RCC (X. D. Liu et al., 2015b). The Von Hippel-Lindau (VHL) tumor suppressor is lost on the short arm of chromosome 3 in the majority of ccRCC (80%). It is well known that loss of VHL leads to induction of the hypoxia inducible factor (HIF), which in turn promotes tumor growth (Gossage and Eisen, 2010). Mikhaylova et al. demonstrated that VHL regulated autophagy in ccRCC. They found that inhibition of autophagy by knockdown of ATG5 resulted in the massive death of VHL(-) cells as compared to VHL(+) cells, indicating that VHL(-) cells could be more dependent on autophagy and therefore more sensitive to inhibition (Mikhaylova et al., 2012). VHL(-) cells also facilitate, in a HIF-independent manner, intracellular nutrients by activation of LC3B-mediated autophagy, which are necessary for tumor growth. Recently, it was reported that VHL mutation in RCC cells induced autophagy by up-regulating the inositol 1,4,5- trisphosphate receptor, type 1 (ITPR1) (Messai et al., 2014).

Folliculin (FLCN) is a tumor suppressor gene that is deficient in Birt-Hogg-Dube syndrome (BHD), a disorder that features renal carcinoma of multiple histological types including hybrid oncocytic RCC, chromophobe RCC, oncocytoma, multiple and bilateral clear cell RCC (Verine et al., 2010). FLCN protein expression is reduced in ccRCC following loss of VHL, and it predicts poor prognosis of RCC (Schmidt et al., 2005). Previously, Bastola et al. illustrated that FLCN promoted autophagy processes by activating the mTORC1 activity in ccRCC cell lines. This indicates that FLCN contributes to the tumor-suppressing effect of VHL (Bastola et al., 2013). Subsequently, Zhang et al. found that paclitaxel-induced autophagy prevented the apoptosis of FLCN-deficient RCC cells. Thus, paclitaxel combined with autophagy inhibitors might be an effective treatment for FLCN-deficient RCC (Zhang et al., 2013).

P53 is up-regulated in tumor tissues, where it may inhibit tumor progression via autophagy. Recent studies have shown that p53 has dual effects in autophagy depending on the subcellular localization. Nuclear p53 facilitates autophagy whereas cytoplasmic p53 inhibits autophagy (Tang et al., 2015; White, 2016). Autophagy also regulates p53. Previously, Kang and Ku et al. found that, in RCC cell lines, transglutaminase 2 (TGase 2), an enzyme regulating covalent crosslinking between protein glutamine and lysine residues, cross-linked p53 into the autophagosome, thereby down-regulating p53 (J. H. Kang et al., 2016; Ku et al., 2013). After the treatment of chloroquine (CQ) and MG132, the binding of p53 with TGase 2 and p62 was potentiated (Ku et al., 2013).

A DNA senor in the cytoplasm that could bind to double-stranded DNA (dsDNA) to cause inflammatory cell death, called pyroptosis (Rathinam et al., 2010), is absent in the tumor suppression melanoma 2 (AIM2). AIM2 enhances the expression of autophagy-related genes *in vitro* and *in vivo*. The low expression levels of AIM2 in RCC reduce autophagy and promote tumorigenesis (Chai et al., 2018).

### Autophagy-Related lncRNAs in RCC

Long non-coding RNAs (lncRNAs) are a class of RNAs that are more than 200 nucleotides in length and could not be translated into proteins (Hong et al., 2017). The silencing of a lncRNA, known as the HOXA transcript at the distal tip (HOTTIP), can induce autophagy by increasing a multitude of autophagy-related genes including Beclin1, LC3B, and LAMP2 through the PI3K/Akt/Atg13 signaling pathway (Su et al., 2019). A recent study by Shao et al. suggested that the lncRNA, a Secretory Carrier Membrane Protein 1 (SCAMP1), inhibited RCC tumorigenesis through activating autophagy in RCC cells (Shao et al., 2019).

### Autophagy-Related Signaling Pathways in RCC

#### PI3K/Akt/mTOR Pathway

Even though there are two forms of mTOR in mammals, most studies only pay attention to mTOR1 because of its sensitivity to rapamycin. The activation of the PI3K/Akt pathway enhances cell survival by inhibiting apoptosis and promoting cell cycle progression by activating mTORC1, thereby inhibiting autophagy (Ren et al., 2016; Deng et al., 2018). For example, Hongyan et al. found that NVP-BEZ235, a novel dual PI3K/mTOR inhibitor, induced cell apoptosis and autophagy in RCC (H. Li et al., 2013). Furthermore, Antonaci et al. indicated that dimethyl sulfide (DMS) induced autophagy in Caki-1 cells through PI3K/AKT/mTOR/p70S6K pathways (Antonaci et al., 2019). Sunitinib blocking the Akt/mTOR/p70S6K pathway has resulted in autophagy activation *in vivo* and *in vitro* (M. L. Li et al., 2018). Deng et al. also reported that sinomenine induced cell apoptosis and autophagy in ACHN cells by making the PI3K/Akt/mTOR signaling pathway inactive (F. Deng et al., 2018). These results suggest that the PI3K/Akt/mTOR signaling pathway and autophagy are important avenues for the prevention and treatment of RCC.

### AMPK/mTOR Pathway

During metabolic stress, high AMP/ATP ratio adenosine activates monophosphate-activated protein kinase (AMPK). AMPK then inhibits mTOR by activating the TSC1/TSC2 protein heterodimer, which results in autophagy activation (Kim et al., 2011). In addition, the AMPK pathway modulates autophagy via an alternative mechanism, in which AMPK stimulates ULK1 and facilitates autophagy due to the phosphorylation of Ser317 and Ser777 (Kim et al., 2011). A recent study found that silibinin repressed the phosphorylation levels of mTOR, increasing the level of AMPK, and markedly promoting the expression of the autophagy marker LC3-Ⅱ. Moreover, the effects of silibinin on mTOR and autophagy were reversed by compound C, a pharmacological inhibitor of AMPK (F. Li et al., 2015). It has also been reported that curcumin has activated autophagy in 786- O and ACHN (5 and 20 μM) via the AMPK signaling pathway (Q. Deng et al., 2018). Resveratrol also induced autophagy of Ketr-3 cells by activating p53/AMPK/mTOR leading to apoptosis of RCC cells (Q. Liu et al., 2018). Among these, the AMPK/mTOR pathway is the primary regulator of autophagy in RCC.

### The Dual Roles of Autophagy in RCC

Although evidence that autophagy regulates both cell survival and death is available, it is not clear why autophagy has dual effects.

Activation of autophagy suppresses tumors by eliminating dysfunctional proteins and damaged cellular organelles and maintaining host defenses (Liang and Jung, 2010). Reduced and aberrant expression of autophagy genes and proteins may affect various aspects of RCC pathology. It has been reported that the cytoprotective enzyme heme oxygenase-1(HO-1) down-regulated autophagy-related proteins Beclin-1 and LC3B-Ⅱ in renal cancer cells (Banerjee et al., 2012). Wang et al. found that activation of autophagy by Atg7 and LC3-Ⅱ overexpression suppressed cell proliferation in 786-O, 769-P, OS-RC-2, ACHN human RCC cell lines *in vivo* and *in vitro* (Wang et al., 2018)*.*

On the other hand, autophagy protects some tumor cells against low-oxygen conditions and nutrient deprivation, which are the main characteristics of tumor microenvironments (Katheder et al., 2017). In VHL-deficient RCC cells, EPAS1, a type of hypoxia-inducible factor, is not degraded but accumulated targeting ITPR1. Meanwhile, ITPR1 regulates sensing a yet undefined signal derived from NK cells to activate autophagy. Activation of autophagy in RCC cells results in the degradation of NK-derived granzyme B (GZMB) which compromises the NK-mediated killing of tumor cells (Messai et al., 2014; Messai et al., 2015). It has also been proven that ITPR1 regulates the NK-mediated killing of RCC cells through the activation of autophagy (Messai et al., 2015). Researchers have reported that various chemotherapeutic drugs for RCC may increase the autophagic flux of RCC cells, and chemotherapeutic drugs combined with autophagy inhibitors may be more effective in controlling RCC progression. For example, temsirolimus (TEM), an mTOR inhibitor, showed good performance on advanced RCC (Voss et al., 2011). However, the effect of TEM is transient in most patients. A study showed that autophagy had protective mechanisms in the regulation of resistance to TEM in RCC (Singla and Bhattacharyya, 2017; Chow et al., 2020). Moreover, the inhibition of autophagy with CQ increased the risk of TEM-induced cell death (Singla and Bhattacharyya, 2017). The Kringle 1 domain of human hepatocyte growth factor (HGFK1) was found to enhance the anti-tumor activities of sorafenib and reverse resistance to this drug in RCC via the inhibition of autophagy (Gao et al., 2019). Recently, a phase I/II trial in patients with RCC showed that the autophagy inhibition achieved by hydroxychloroquine enhanced the anti-tumor effects of mTOR inhibitors (Haas et al., 2019). The PI3K/mTOR dual inhibitor NVP-BEZ235 (50, 100 or 500 and 1,000 nM) induced 786-0 cells apoptosis and autophagy by increasing LC3-Ⅱ and decreasing p62. Autophagy inhibitors significantly potentiated the anticancer effect of NVP-BEZ235 (H. Li et al., 2013).

### Autophagy and Therapy in RCC

#### Autophagy Inducers as Treatments for RCC

Studies have reported that the activation of autophagy may exert therapeutic effects against RCC, some of which are shown in Table 1. Mutations and/or inactivation of the VHL tumor suppressor gene exist in most RCC and are relevant to poor prognosis. Turcotte et al. (Turcotte et al., 2008) designed a compound, STF-62247, which selectively targeted VHL-deficient cells *in vitro* and *in vivo.* The compound enhanced autophagy by influencing Golgi trafficking and PI3K passage in VHL-deficient cells. VHL is a significant negative regulator of HIF-a. They found that STF-62247 induced autophagy in a HIF-independent manner. The VHL-deficient RCC was more sensitive to STF-62247 compared to cells with wild-type VHL, establishing a synthetic lethal situation due to the combining drug treatment and VHL deficiency. In addition, STF-62247 increased the radiosensitivity of VHL-deficient RCC cells and 786-O cells by inducing autophagy (Anbalagan et al., 2012). Ubenimex induced RCC cell death by upregulating autophagy, as evidenced by increased LC3B (S. Liu et al., 2015c). (*R*)-goniothalamin and (*S*)-goniothalamin, a pair of styryllactone enantiomer extracted from plants of the genus *Goniothalamus,* induced the death of human kidney cancer cells (786-0) primarily by enhancing the expression of LC3B (de Fatima et al., 2008). Silibinin is a flavonoid derived from the seeds of milk thistle. The study showed that autophagy induction by silibinin positively contributing to its anti-metastatic capacity in human RCC cells (ACHN and 786-O) by increasing the expression of LC3-II and enrichment of autophagolysosome vacuoles via the AMPK/mTOR pathway (F. Li et al., 2015). Resveratrol induced Ketr-3 cells apoptosis by triggering ATG5 and ATG7 expression through p53-mediated AMPK/mTOR signaling (Q. Liu et al., 2018). In ACHN cell lines, sinomenine, an isoquinoline extracted from *Sinomenium acutum*, inhibited RCC progression by inducing autophagy via Beclin1 and LC3-Ⅱ/LC3-Ⅰ up-regulation and p62 down-regulation (F. Deng et al., 2018). Rasfonin (A304), a product of *Talaromyces sp*, activates the death of RCC cells by inducing autophagy, and this effect could be suppressed by the Akt inhibitor (Lu et al., 2015; Sun et al., 2016).

**TABLE 1:** Modulate autophagy compounds.

Compound	In vitro/*in vivo*	Target	Regulate autophagy	Biological role	Refernces
STF-62247	in vitro and *in vivo*	LC3-Ⅱ	Induce	Cell death (+)	Anbalagan et al. (2012)
Ubenimex	in vitro	LC3B	Induce	Cell death (+)	Liu et al. (2015)
(R)-goniothalamin and (S)-goniothalamin	in vitro	LC3B	Induce	Cell death (+)	de Fatima et al. (2008)
Silibinin	in vitro and *in vivo*	LC3-Ⅱ	Induce	Metastasis (−)	F. Li et al. (2015)
Resveratrol	in vitro	ATG5, ATG7	Induce	Apoptosis (+)	Q. Liu et al. (2018)
Sinomenine	in vitro	Beclin1, LC3-Ⅱ/LC3-Ⅰ, p62	Induce	Apoptosis (+)	F. Deng et al. (2018)
Rasfonin	in vitro	LC3-Ⅱ, p62	Induce	Apoptosis (+), necroptosis (+)	Sun et al. (2016)
Chloroquine	in vitro	Deacidifying the lysosome	Inhibit	Apoptosis (+)	Grimaldi et al. (2015)
3-Methyladenine	in vitro	LC3-Ⅱ	Inhibit	Apoptosis (+)	Zhang et al. (2013)
Bafilomycin A1	in vitro	Deacidifying the lysosome	Inhibit	Apoptosis (+)	Zhang et al. (2013)

(+) = increased, (-) = decreased.

### Autophagy Inhibitors for the Treatment of RCC

As discussed in previous sections, autophagy may be a survival mechanism in most cellular contexts as it prevents or delays cancer cell death (Altman and Rathmell, 2012). In cancer cells exposed to stress stimuli such as hypoxia, nutrient deficiency, and chemotherapy, autophagy is activated as a protective mechanism to maintain the survival of cancer cells (Mathew and White, 2011). It has been found that autophagy induced by heteronemin partially antagonized cytotoxicity and apoptotic signaling in human renal carcinoma A498 cells (Wu et al., 2015). Chauhan et al. demonstrated that autophagy inhibitors enhanced apoptosis in A498 cells (Chauhan et al., 2017). Therefore, autophagy is considered a novel therapeutic target (Table 1).

Grimaldi et al. identified that chloroquine (CQ) improved the efficacy of everolimus and sunitinib by down-regulating autophagy in RCC cells (Grimaldi et al., 2015; M. L.; Li et al., 2018). Similar to CQ, hydroxychloroquine (HCQ) inhibits autophagy via deacidifying the lysosome to block its fusion with autophagosome (Levy et al., 1997). Four clinical trials of autophagy are currently ongoing to test the performance single HCQ or its combination with other drugs on RCC (Levy et al., 1997). Elsewhere, it was found that paclitaxel-activated apoptosis induced by the inhibition of autophagy with 3-Methyladenine (3-MA) and bafilomycin significantly enhanced in FLCN-deficient RCC cells (Zhang et al., 2013). Interestingly, bafilomycin A1 was found to block autophagy by inhibiting the fusion of autophagosome and lysosome. Hence, a combination of autophagy inhibitors and other therapies may effectively control RCC.

## Conclusion

Autophagy is modulated by multiple intracellular processes in varied stressful conditions, such as during organelle dysfunction, nutrient deprivation, and anticancer therapy. The data reviewed here show that autophagy has a dual role in the initiation, progression, treatment, and drug resistance of RCC. In general, autophagy suppresses tumor development by eliminating oxidative stress, maintaining genomic stability, and reducing dysfunctional proteins in RCC. However, chemotherapies targeting RCC activate autophagy inducing drug tolerance and hence promote tumor progression. This indicates that drugs that restrain autophagy could be effective treatments for RCC. In other words, chemotherapies combined with autophagy inhibitors may be more effective, especially for chemotherapy-resistant RCC. Studies have documented that autophagy regulators may regulate the AMPK/mTOR and PI3K/Akt/mTOR signaling pathways to confer therapeutic effects in RCC. Further research is needed to reveal the clinical significance of autophagy inhibitors and activators in RCC.

How to control and exploit autophagy for diagnostics and treatment in RCC needs further discussion. We believe that further research on autophagy will lead to the design of important therapeutic strategies for the treatment of RCC.

**FIGURE 1:**
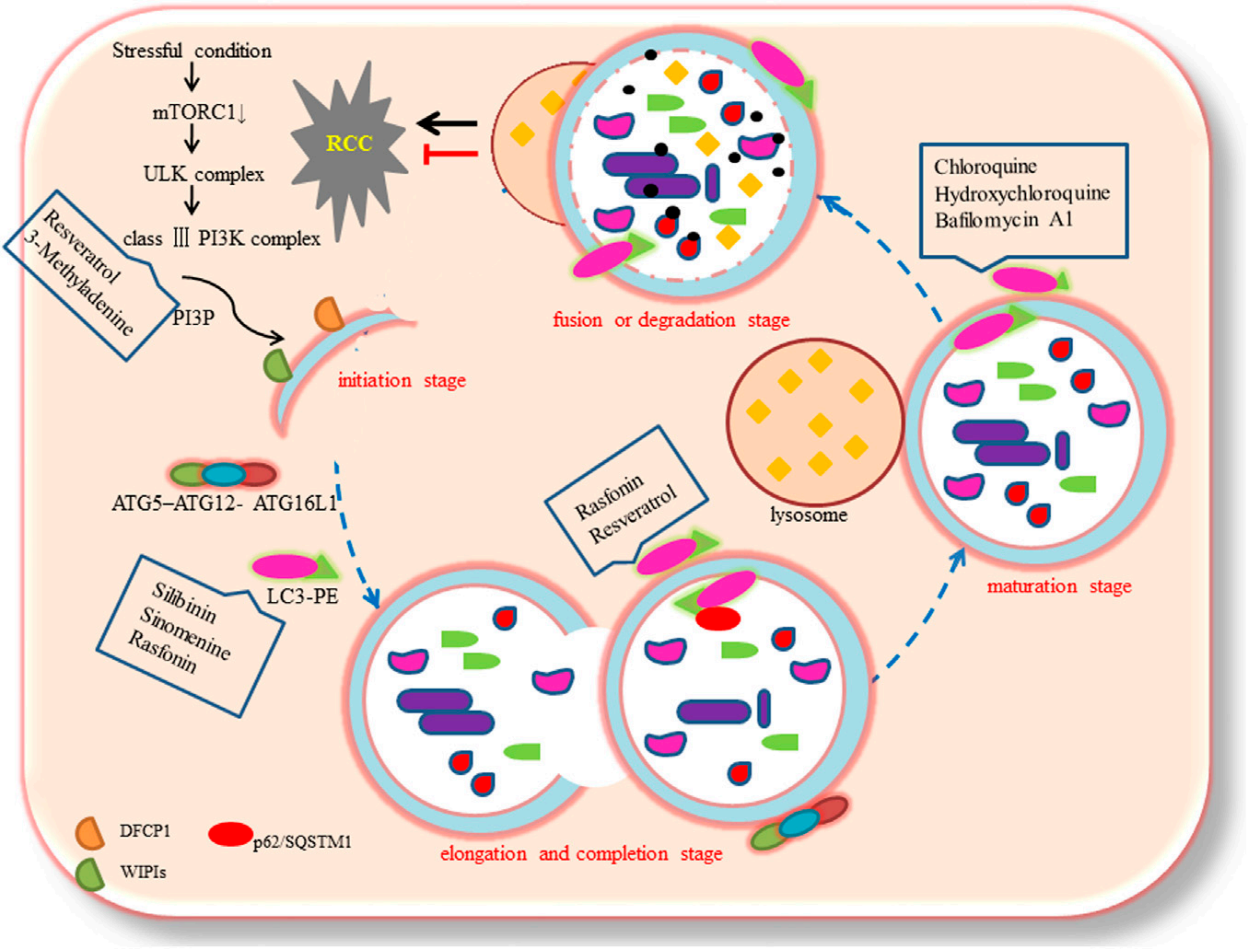
The process of autophagy.
